# Social Determinants of Community Health Services Utilization among the Users in China: A 4-Year Cross-Sectional Study

**DOI:** 10.1371/journal.pone.0098095

**Published:** 2014-05-22

**Authors:** Yanhong Gong, Xiaoxv Yin, Yunxia Wang, Yongbin Li, Geng Qin, Liqun Liu, Wei Zhou, Fujian Song, Xiaoxin Dong, Shiyi Cao, Chen Yang, Huajie Yang, Jun Xie, Junan Liu, Zuxun Lu

**Affiliations:** 1 Department of Social Medicine and Health Management, School of Public Health, Tongji Medical College, Huazhong University of Science and Technology, Wuhan, Hubei, P. R. China; 2 Department of Maternal and Child Health Care and Community Health, National Health and Family Planning Commisson of the People's Republic of China, Beijing, P. R. China; 3 Norwich Medical School, Faculty of Medicine and Health Science, University of East Anglia, Norwich, United Kingdom; Johns Hopkins Bloomberg School of Public Health, United States of America

## Abstract

**Background:**

To identify social factors determining the frequency of community health service (CHS) utilization among CHS users in China.

**Methods:**

Nationwide cross-sectional surveys were conducted in 2008, 2009, 2010, and 2011. A total of 86,116 CHS visitors selected from 35 cities were interviewed. Descriptive analysis and multinomial logistic regression analysis were employed to analyze characteristics of CHS users, frequency of CHS utilization, and the socio-demographic and socio-economic factors influencing frequency of CHS utilization.

**Results:**

Female and senior CHS clients were more likely to make 3–5 and ≥6 CHS visits (as opposed to 1–2 visits) than male and young clients, respectively. CHS clients with higher education were less frequent users than individuals with primary education or less in 2008 and 2009; in later surveys, CHS clients with higher education were the more frequent users. The association between frequent CHS visits and family income has changed significantly between 2008 and 2011. In 2011, income status did not have a discernible effect on the likelihood of making ≥6 CHS visits, and it only had a slight effect on making 3–5 CHS visits.

**Conclusion:**

CHS may play an important role in providing primary health care to meet the demands of vulnerable populations in China. Over time, individuals with higher education are increasingly likely to make frequent CHS visits than individuals with primary school education or below. The gap in frequency of CHS utilization among different economic income groups decreased from 2008 to 2011.

## Introduction

The primary health care (PHC) system was once inexpensive to the users of the PHC and played an important role in improving the population's health in China [1], [2]. The economic reforms that have transformed China since 1978 unleashed a boom in economic changes, but they also had negative effects on the PHC system [3], [4]. The disparity between the increasing demand for and inadequate supply of safe and effective healthcare, the escalating medical costs, and the absence of insurance coverage made the public identify the problem of it being “too difficult and too expensive to see a doctor” as one of the key public policy issues [5], [6]. In order to resolve this problem and supply affordable and equitable PHC for all, the latest round of healthcare reform was initiated in 2009 [7], [8]. China's long-term strategy of the healthcare reform involves building a strong delivery system based on PHC [7], [8].

Since the healthcare reform, government funds invested in healthcare have increased substantially, rising from ¥3.59 billion (US$0.52 billion) in 2008 to ¥7.46 billion (US$1.15 billion) in 2011 [9]. About 30% of the government funds are allocated to the building of supply-side infrastructure and training of PHC providers [7]. The number of community healthcare service (CHS) institutions increased dramatically between 2008 and 2011. Total number of CHS institutions was 24,260 in 2008, 27,308 in 2009, 32,739 in 2010, and 32,860 in 2011 [9]. During this time, the number of healthcare workers per CHS institutions increased as well [9]. Although a large amount of money was spent in improving PHC, utilization of PHC did not increase significantly. No shift in the flow of patients from high-level health institutions to PHC facilities was recorded between 2008 and 2011 [8]–[10]. Previous research showed that 64.8% of outpatients and 76.8% of inpatients with chronic diseases who sought health services in high-level hospitals could also access PHCs to meet their health needs in China [11].

Thus, providing a means of increasing PHC utilization is an important challenge in China. To improve PHC utilization, it is necessary to know about its determinants, which include factors influencing the user's decision to make initial contact with PHC service and frequency of PHC utilization. The aim of our study was to characterize demographic profiles of CHS clients and identify socio-demographic and socio-economic factors determining the frequency of CHS utilization.

## Methods

### Ethics statement

The study protocol and the questionnaire were approved by the Research Ethics Committee of Huazhong University of Science and Technology, Wuhan, China. All participants read a statement that explained the purpose of the survey and gave written informed content before being involved in the investigation.

### Data source and sampling

Nationwide cross-sectional surveys that aimed to monitor and assess the development of CHS in China were conducted in 2008, 2009, 2010, and 2011, which were titled “the Chinese Community Health Service System Development”. A multistage sampling method was employed in our study. First, cities across China were divided into three groups: developed eastern cities, least-developed western cities, and “middle” cities between the other two in terms of development. Thirty-five cities were selected according to geographic regions, economic and political characteristics, city size, and the development level of CHS. Second, the cities were further divided into city districts. Two community health centers and two community health stations were randomly selected in every district of the sampled cities, except in Xi'an City. Almost all CHS facilities in Xi'an City were enrolled because the local Health Bureau intended to collect census data of the CHS. Furthermore, interviewers began to collect information from CHS clients. A survey was conducted at the exit of the CHS institutions, based on convenience sampling. Thirty outpatients from each community health center and twenty from each community health station were interviewed. All interviewers received adequate training to optimize the reliability of the survey.

During our investigation, 93,933 CHS clients were recruited, of whom 5,083 clients refused to participate. Additionally, 721 questionnaires were discarded because of missing data or logical error. Finally, a total of 88,119 eligible questionnaires remained, with an overall response rate of 93.82% of those asked to participate. Of the 88,119 participants, 86,116 CHS clients ages 15 or above were included in the analysis based on their ability to act independently.

### Measures

The dependent variable was the frequency of CHS utilization (i.e. “Over the past 12 months, how many times did you utilize the CHS?”). The question was closed-ended with six response options (1 time to 6 times or more). CHS included medical services and public health services.

The independent variables included socio-demographics characteristics (gender, age), socio-economic status [educational level, employment status, and household income per capita (HIPC)], type of medical insurance coverage, and travel time to the visited CHS facilities on foot (not collected in 2008). To simplify interpretation of the odds ratio (OR), participants' HIPC measures were divided into four classes based on the urban residents' disposable income per capita (URDIPC) of their respective cities from the corresponding year [income level 1 (HIPC<50% URDIPC), income level 2 (50% URDIPC≤HIPC<URDIPC), income level 3 (URDIPC≤HIPC<200% URDIPC) and income level 4 (HIPC≥200% URDIPC)]. Fifty percent of the average or median income is often applied as the low-income line, so individuals who belong to income level 1 can also be regarded as the low-income group. In addition, there are currently three main social medical insurance programs, Urban Employee Basic Medical Insurance (UEBMI), Urban Resident Basic Medical Insurance (URBMI), and New Cooperative Medical Scheme (NCMS), and three nonmainstream ones, Government Medical Insurance (GMI), Labor Medical Insurance (LMI), and Commercial Medical Insurance (CMI), in China.

### Data analysis

All statistical procedures were performed by using the SAS 9.2 statistical software package (SAS Institution Inc., Cary, NC, USA). Specialized SAS procedures for survey sampling were employed. Descriptive analysis was carried out for socio-demographics, socio-economic characteristics, and frequency of CHS utilization. Multinomial logistic regression analysis was used to analyze socio-demographic and socio-economic factors that determined frequency of CHS utilization (3–5 CHS visits or ≥6 CHS visits), with 1–2 CHS visits as the reference category. The primary analysis was based on a study sample not stratified by gender. A second analysis, stratified by gender, was also conducted to test the associations for men and women separately (Table S1 and Table S2). Results from this analysis were not significantly different from those in the primary analysis. Therefore, we present only the results of the primary analysis to simplify interpretation of results. Altman and Bland outlined statistical method to compare the difference between two estimates of the same quantity derived from separate analyses [12]. In our study, we used this method to compare ORs derived from different multinomial logistic regression models. Sampling weights were not used in the analysis because they could not be calculated. For all comparisons, differences were tested using two-tailed tests and *p*<0.05 was considered statistically significant.

## Results

Table 1 presents the basic characteristics of the CHS clients by year; as can be seen, most variables were relatively well balanced by year. The proportion of uninsured people decreased from 19.59% in 2008 to 8.58% in 2011. Of the participants, approximately one-third required more than 15 minutes to get to the visited CHS facilities on foot. Utilization of CHS, measured by the number of CHS visits in a year, is summarized in Table 2. The median of CHS visits was all 3 times in 2008–2011. The proportion of people who had ≥6 visits was 31.29% (2008), 29.62% (2009), 26.88% (2010), and 30.90% (2011).

**Table 1:** Descriptive statistics of sample characteristics between 2008 and 2011.

Variables	2008	2009	2010	2011
**No. of participants**	19375	22151	21820	22770
**Age (Mean±S.D.)**	49.08±17.16	49.49±17.23	49.56±16.79	49.41±17.08
**Gender**				
male	44.26	44.79	42.56	43.31
Female	55.74	55.21	57.44	56.69
**Education**				
Primary school or below	19.92	20.22	19.71	18.20
Junior middle school	27.77	27.74	28.42	28.11
Senior middle school	31.20	30.82	28.70	28.87
College degree or above	21.11	21.22	23.18	24.83
**Employment status**				
Unemployment	7.35	6.47	6.78	5.60
Employment	42.23	42.72	43.17	45.68
Retire	33.79	34.62	32.54	33.83
Others (student, housewife, etc.)	16.63	16.20	17.51	14.89
**Household income per capita**				
Income level 1	18.78	16.34	12.65	12.10
Income level 2	38.58	39.72	35.43	32.33
Income level 3	29.92	30.16	36.74	41.68
Income level 4	12.71	13.79	15.18	13.89
**Insurance**				
Uninsured	19.59	14.22	11.64	8.58
GMI	12.70	9.92	9.00	7.72
UEBMI/LMI	40.65	44.69	45.52	47.49
URBMI	15.62	17.40	17.24	19.37
NCMS	8.51	11.30	15.50	15.99
CMI	2.93	2.47	1.10	0.84
**District**				
Western	31.92	34.19	34.00	32.89
Central	21.34	21.63	22.03	23.77
Eastern	46.74	44.18	43.97	43.34
**Travel time (Mins)**				
<15	-	63.35	64.44	63.34
15+	-	36.65	35.56	36.66

GMI = Government Medical Insurance, UEBMI =  Urban Employee Basic Medical Insurance, URBMI = Urban Resident Basic Medical Insurance, LMI = Labor Medical Insurance, NCMS = New Cooperative Medical Scheme, CMI = Commercial Medical Insurance

**Table 2:** Descriptive statistics of frequency of CHS utilization among CHS users from 2008 to 2011.

Variables	No. of visits in 2008			No. of visits in 2009			No. of visits in 2010			No. of visits in 2011		
	1–2	3–5	≥6	1–2	3–5	≥6	1–2	3–5	≥6	1–2	3–5	≥6
**Total**	33.33	35.39	31.29	32.52	37.86	29.62	38.27	34.86	26.88	35.06	34.04	30.90
**Education**												
Primary school or below	25.54	33.07	41.39	24.46	36.46	39.08	32.42	35.95	31.64	31.75	32.74	35.51
Junior middle school	32.29	35.66	32.05	30.00	39.29	30.71	37.21	34.35	28.44	33.85	33.92	32.23
Senior middle school	36.02	36.32	27.66	35.71	38.67	25.62	37.89	35.60	26.51	35.47	34.90	29.63
College degree or above	38.22	35.65	26.13	38.93	36.11	24.96	45.04	33.57	21.38	38.44	34.14	27.42
**Household income per capita**												
Income level 1	36.25	31.64	32.12	29.28	39.11	31.61	37.15	35.59	27.26	37.17	32.85	29.99
Income level 2	32.22	35.95	31.83	30.92	38.83	30.25	37.33	34.94	27.73	34.88	34.53	30.60
Income level 3	33.35	37.32	29.33	35.42	36.71	27.88	38.63	34.27	27.11	33.91	34.04	32.04
Income level 4	31.92	35.57	32.50	34.05	35.64	30.31	39.31	35.86	24.83	36.74	35.54	27.72

CHS = community health service.

Adjusted multinomial logistic regression analyses were used to examine the association between socio-demographic and socio-economic factors and the frequency of CHS utilization (Table 3). The results indicated that age was positively associated with making both 3–5 and ≥6 CHS visits, compared to 1–2 visits. Female CHS clients were more likely to make 3–5 and ≥6 CHS visits than were their male counterparts.

**Table 3:** Multinomial logistic regression for the association with frequency of CHS utilization among CHS users.

Variables	2008		2009		2010		2011	
	3–5 visits	≥6 visits	3–5 visits	≥6 visits	3–5 visits	≥6 visits	3–5 visits	≥6 visits
**Gender (ref = male)**	1.12(1.07–1.18)***	1.37(1.28–1.47)***	1.14(1.12–1.16)***	1.30(1.24–1.36)***	1.09(1.08–1.11)***	1.30(1.26–1.33)***	1.11(1.10–1.13)***	1.27‡(1.25–1.30)***
**Age** §	1.11(1.09–1.14)***	1.33(1.30–1.35)***	1.14(1.11–1.16)***	1.40(1.38–1.42)***	1.10(1.07–1.13)***	1.25(1.19–1.30)***	1.11(1.09–1.12)***	1.29‡(1.26–1.32)***
**Education (ref = primary school or below)**								
Junior middle school	0.93(0.88–1.00)*	0.94(0.90–0.99)*	0.99(0.92–1.07)	0.96(0.86–1.07)	0.94(0.91–0.98)**	1.11(1.02–1.20)*	1.09†(1.05–1.14)***	1.26‡(1.21–1.30)***
Senior middle school	0.88(0.79–0.97)**	0.88(0.84–0.92)***	0.92(0.85–0.99)*	0.86(0.78–0.95)**	1.02(0.99–1.06)	1.28(1.20–1.36)***	1.14†(1.10–1.18)***	1.41‡(1.33–1.50)***
College degree or above	0.81(0.75–0.88)***	0.86(0.77–0.97)*	0.85(0.78–0.93)***	0.98(0.86–1.12)	0.90(0.86–0.93)***	1.15(1.05–1.26)**	1.09†(1.05–1.13)***	1.48‡(1.43–1.54)***
**Employment status (ref = unemployment)**								
Employment	1.00(0.96–1.05)	0.95(0.84–1.08)	0.99(0.95–1.04)	0.90(0.85–0.94)***	0.97(0.92–1.03)	0.66(0.61–0.72)***	1.16†(1.12–1.21)***	1.04(0.95–1.14)
Retire	1.12(1.04–1.19)**	1.85(1.65–2.08)***	1.20(1.11–1.30)***	1.66(1.51–1.82)***	1.04(0.92–1.18)	1.24(1.08–1.42)**	1.26†(1.17–1.36)***	1.91(1.68–2.18)***
Others (student, housewife)	0.85(0.81–0.89)***	1.28(1.08–1.52)**	0.86(0.80–0.92)***	1.04(0.99–1.09)	0.90(0.87–0.94)***	1.09(1.04–1.14)***	1.07†(0.98–1.16)	1.41(1.32–1.51)***
**Household income per capita (ref = income level 1)**								
Income level 2	1.30(1.24–1.37)***	1.11(1.03–1.20)**	1.18(1.12–1.25)***	1.14(1.09–1.19)***	1.01(0.92–1.11)	0.98(0.86–1.12)	1.10†(1.00–1.20)*	1.00‡(0.97–1.03)
Income level 3	1.42(1.29–1.55)***	1.15(1.09–1.21)***	1.11(1.03–1.19)**	1.13(1.09–1.17)***	1.01(0.93–1.09)	1.00(0.87–1.14)	1.16†(1.05–1.28)**	1.08(0.99–1.17)
Income level 4	1.53(1.37–1.70)***	1.55(1.42–1.69)***	1.19(1.15–1.24)***	1.51(1.40–1.63)***	1.09(0.98–1.22)	0.93(0.71–1.21)	1.16†(1.03–1.31)*	0.97‡(0.86–1.09)
**Insurance (ref = uninsured)**								
GIS	0.89(0.82–0.97)**	1.38(1.17–1.63)***	1.04(0.97–1.12)	1.40(1.29–1.52)***	0.97(0.92–1.03)	1.34(1.18–1.51)***	0.87(0.82–0.93)***	1.27(1.13–1.43)***
UEBMI/LMI	1.02(0.96–1.08)	1.45(1.29–1.62)***	1.18(1.11–1.26)***	1.53(1.43–1.63)***	1.22(1.18–1.27)***	1.49(1.30–1.70)***	1.13†(1.06–1.20)***	1.68(1.49–1.90)***
URBMI	0.95(0.91–0.99)*	1.11(0.96–1.28)	1.22(1.14–1.31)***	1.3(1.140–1.49)***	1.30(1.25–1.34)***	1.21(1.12–1.32)***	1.24†(1.16–1.33)***	1.67‡(1.53–1.82)***
NCMS	1.13(1.03–1.24)**	0.72(0.61–0.84)***	1.29(1.15–1.45)***	0.90(0.81–1.00)*	1.33(1.25–1.43)***	1.14(1.04–1.24)**	1.16(1.06–1.27)***	1.42‡(1.30–1.54)***
CMI	0.93(0.86–1.00)*	1.11(0.91–1.35)	0.90(0.78–1.05)	0.56(0.50–0.63)***	0.72(0.63–0.82)***	0.96(0.76–1.21)	1.20†(0.96–1.51)	1.55‡(1.33–1.80)***
**District (ref = western)**								
Central	1.08(0.97–1.20)	0.94(0.76–1.16)	0.86(0.81–0.91)***	0.64(0.62–0.66)***	0.78(0.74–0.83)***	0.57(0.53–0.62)***	1.10(1.02–1.20)*	0.78(0.66–0.94)**
Eastern	0.97(0.86–1.10)	1.55(1.11–2.16)*	1.01(0.87–1.17)	1.43(1.15–1.78)**	0.96(0.90–1.04)	1.47(1.21–1.80)***	1.13†(1.04–1.23)**	1.92(1.53–2.42)***
**Travel time(ref = 15+ Mins)**								
<15	-	-	1.02(0.99–1.05)	1.30(1.19–1.41)***	0.98(0.93–1.02)	1.34(1.28–1.40)***	0.93µ(0.90–0.96)***	1.72#(1.61–1.84)***

^*^P<0.05;

^**^P<0.01;

^***^P<0.0001 (two-tailed test);

§ The odds ratios of age represent the change in the odds when the variable age is increased by ten years;

† The difference between the odd ratios of making 3–5 CHS visits in 2008 and 2011 was significant;

‡ The difference between the odd ratios of making ≥6 visits in 2008 and 2011 was significant;

µ The difference between the odd ratios of making 3–5 CHS visits in 2009 and 2011 was significant;

# The difference between the odd ratios of making ≥6 visits in 2009 and 2011 was significant.

CHS = community health service, GMI = Government Medical Insurance, UEBMI =  Urban Employee Basic Medical Insurance, URBMI = Urban Resident Basic Medical Insurance, LMI = Labor Medical Insurance, NCMS = New Cooperative Medical Scheme, CMI = Commercial Medical Insurance

CHS clients with higher education showed a lower likelihood of making 3–5 and ≥6 CHS visits (as opposed to 1–2 visits) than those with less education during the first two years; however, in 2011 they showed a higher likelihood of making 3–5 and ≥6 visits. Comparing the ORs of each subgroup between 2008 and 2011 illustrated that the relationship between education and making 3–5 CHS visits changed significantly over time (Table 3; details in Table S3 and S4). The same pattern occurred in the relationship between education and making ≥6 CHS visits.

The associations between economic status and frequency of CHS visits have changed from 2008 to 2011. For CHS clients, participants with higher income were more likely than the clients with the lowest income to make 3–5 and ≥6 CHS visits at the first two years. In 2010 and 2011, the socio-economic status did not have a discernible effect on utilizing CHS ≥6 times. Comparing the ORs of each subgroup between 2008 and 2011 illustrated that the ORs decreased significantly in almost all subgroups (Table 3; details in Table S3 and S4).

The effect of medical insurance on the probability of frequent CHS visits was obvious, but the effect of different types of medical insurance differed. Across the four years, CHS clients insured by GMI or UEBMI/LMI were more likely to take ≥6 CHS visits than their uninsured counterparts were. In the beginning, CHS clients insured by URBMI, NCMS or CMI showed a lower likelihood of making 3–5 or ≥6 CHS visits than their uninsured counterparts; while in 2011, CHS clients insured by URBMI, NCMS or CMI all had a greater odd of making 3–5 or ≥6 visits. The impact of URBMI, NCMS, and CMI on the probability of making ≥6 visits changed significantly from 2008 to 2011 (Table 3; details in Table S4).

The association between geographic regions (eastern, central, and western) and frequency of CHS utilization was significant. Eastern CHS consumers had greater odds to make ≥6 visits compared to 1–2 visits than did the western CHS clients. Furthermore, western CHS users had a greater likelihood of making ≥6 visits compared to 1–2 visits than did their central counterparts.

In addition, travel time to the visited CHS facilities on foot was an important factor determining the frequency of CHS utilization. CHS users who took less than 15 minutes to reach the visited CHS facilities were more likely to make ≥6 visits compared to 1–2 visits than were people taking more than 15 minutes.

## Discussion

This study analyzed the frequency of accessing CHS resources. Results showed that probability of frequent visits to CHS facilities was greater for women, for seniors, and for those retired. As time went on, the difference in making more frequent CHS visits among different economic income groups lessened. In 2011, income status did not have a discernible effect on the likelihood of making ≥6 visits, and it only has a slight effect on making 3–5 CHS visits. These results indicated that CHS may play an important role in providing PHC to meet the demands of vulnerable populations (e.g. females, the older population, and the low-income group). Higher use of PHC services by females and older adults has been reported in previous studies [13]–[15]. It has been argued that females and older adults have more health needs and that females are more aware of health matters than are males [14], [16].

There are obvious variations regarding the relationship between education and frequency of CHS utilization. In 2008 and 2009, people with higher education were less likely to make frequent CHS visits, which may be because they distrusted the quality of PHC, and therefore bypassed it to go to over-crowded hospitals [17]. Beginning in 2010, individuals with higher education were increasingly likely to make frequent visits to CHS. Previous studies discovered a lower use of PHC by people with lower levels of education, which could be attributed to the lack of information on matters concerning their health [18]. We suggested that increased utilization of CHS by people with higher education is due to a series of policies implemented during the healthcare reform. Increased government subsidies for basic construction, purchasing equipment, and training providers of PHC improved the quality of CHS, which enhanced public confidence in CHS. Further, PHC providers have been delivering a defined package of basic public health services for the population for free since 2009. Individuals with higher education may have been familiar with the relevant policies earlier than the less educated ones, which may have caused them to make more use of CHS, especially for basic public health services. The unequal CHS utilization by educational level suggests it is necessary to strengthen the marketing and publicity of relevant policies.

In our study, household income was an important determinant of frequency of CHS utilization, which was not consistent with previous research that suggested that income had no significant effect on CHS utilization [13], [18]–[20]. However, the effects decreased dramatically from 2008 to 2011. This change may reflect the benefits of the improved health system. In 2009, URBMI was established in urban areas in China and the number of CHS institutions registered with social medical insurance increased rapidly. In addition, the essential medicines program was executed nationwide at the PHC level in 2009, ensuring the availability of effective medications that satisfy high priority public health care needs. These strategies reduced financial barriers and promoted equitable access to CHS for low-income individuals. This explanation was supported by the survey question on overall satisfaction with the price of CHS. Results indicated that the satisfaction for low-income group increased from 73.61% in 2008 to 88.06% in 2011.

Medical insurance increased the probability of frequent visits to CHS, which is consistent with previous studies' results [20]. The gap between the ORs of different types of medical insurance decreased, with closer probabilities of more frequent CHS visits, as a result of improved benefit packages and lessened inequality in reimbursement rates under various programs. Therefore it is necessary to make great efforts not only in expanding insurance coverage and benefit packages provided by medical insurance companies, but also in lessening the gap in benefit packages among different types of insurance [21].

The effect of distance to CHS institutions on frequency of CHS utilization was significant, which was consistent with previous studies [22]–[26]. Distance to primary healthcare facilities was an important aspect in the availability of CHS. In order to increase CHS utilization, CHS institutions should be strategically placed to guarantee convenience in access to CHS.

Our results indicated clear regional variations in the frequency of CHS utilization. Middle and western CHS clients were less likely to make frequent use of CHS than were eastern ones. Regional variations may be attributed to unequal distribution of health resources. The eastern provinces possess better and healthier resources than do middle and western ones. For example, the number of physicians per 1000 people in eastern, middle, and western China in 2006 was 1.81, 1.39, and 1.40, respectively; the number of nurses per 1000 people was 1.38, 0.99, and 0.90, respectively [27]. Additionally, considering that western China receives more financial aid from the central government, health resources in western China can be comparable to or even richer than resources in the middle of China. The health expenditure per capita in the eastern, middle, and western regions of China in 2005 was ¥100.56, ¥49.04, and ¥74.86, respectively [27]. In consideration of unequal distribution of health resources across regions, the State Council released a health reform guideline in 2009, but it will take some time to reduce such an inequality.

## Limitations

This study has a few limitations that must be acknowledged. First, the results cannot be generalized to the whole population of CHS users because of selection bias and a lack of information concerning the sampling frame, both resulting from our use of convenience sampling. For instance, individuals who made more visits to CHS facilities were more likely to be recruited. However, our study was a nationwide survey and the sample size was enormous, which can somewhat counter this limitation. Second, the study only identifies the socio-demographic and socio-economic factors affecting the frequency of CHS utilization among CHS clients. It is estimated that around 40% have not made use of CHS [28], making it important to explore the factors that aid or inhibit CHS utilization in the general Chinese population. Third, both individual health status and psychological factors have not been taken into consideration, which are other important determinants of the utilization of health services.

## Conclusion

In summary, CHS may play an important role in providing primary health care to meet the demands of the vulnerable populations in China. Over time, individuals with higher education are increasingly likely to make frequent visits to CHS facilities than individuals with primary school education level or below, which implies an inequality in the frequency of CHS utilization resulting from differences in education levels. The gap in the frequency of CHS utilization among different economic income groups lessened from 2008 to 2011.

## Supporting Information

Table S1 **Multinomial logistic regressions for the association with frequency of CHS utilization among male CHS users.**
(DOC)

Table S2 **Multinomial logistic regressions for the association with frequency of CHS utilization among female CHS users.**
(DOC)

Table S3 **Comparison of odds ratios of making 3–5 CHS visits in 2008 and 2011.**
(DOC)

Table S4 **Comparison of odds ratios of making ≥6 CHS visits in 2008 and 2011.**
(DOC)
