# Supplementary material for: Social Determinants of Community Health Services Utilization among the Users in China: A 4-Year Cross-Sectional Study
Source: PLoS One. 2014 May 22;9(5):e98095. doi: 10.1371/journal.pone.0098095 (PMC4031144; doi:10.1371/journal.pone.0098095)
Supplement: Table S1 — Multinomial logistic regressions for the association with frequency of CHS utilization among male CHS users. (DOC) [file pone.0098095.s001.doc]

**Table S1 Multinomial logistic regressions** for the association with frequency of CHS utilization among male CHS users

| **Variables** | **2008** | | **2009** | | **2010** | | **2011** | |
| --- | --- | --- | --- | --- | --- | --- | --- | --- |
|  | **3–5 visits** | **≥6 visits** | **3–5 visits** | **≥6 visits** | **3–5 visits** | **≥6 visits** | **3–5 visits** | **≥6 visits** |
| **Age^§^** | 1.13(1.11-1.14)^***^ | 1.40(1.38-1.42)^***^ | 1.17(1.15-1.19)^***^ | 1.50(1.47-1.53)^***^ | 1.09(1.06-1.12)^***^ | 1.26(1.20-1.32)^***^ | 1.12(1.11-1.12)^***^ | 1.38(1.32-1.45)^***^ |
| **Education (ref=primary school** **or below)** | | | | | | | | |
| Junior middle school | 0.95(0.89-1.02) | 1.03(0.97-1.10) | 1.01(0.90-1.14) | 0.98(0.85-1.14) | 0.92(0.84-1.02) | 1.13(0.99-1.28) | 1.13^†^(1.02-1.24)^*^ | 1.28^‡^(1.24-1.32)^***^ |
| Senior middle school | 0.89(0.78-1.02) | 0.99(0.90-1.08) | 0.85(0.75-0.97)^*^ | 0.83(0.73-0.95)^**^ | 1.04(0.96-1.13) | 1.37(1.18-1.60)^***^ | 1.22^†^(1.17-1.27)^***^ | 1.53^‡^(1.39-1.69)^***^ |
| College degree or above | 0.83(0.71-0.96)^*^ | 1.02(0.89-1.17) | 0.77(0.65-0.92)^**^ | 0.96(0.81-1.13) | 0.81(0.73-0.91)^***^ | 1.07(0.87-1.30) | 1.13^†^(1.09-1.17)^***^ | 1.49^‡^(1.36-1.62)^***^ |
| **Employment status (ref=unemployment)** | | | | | | | | |
| Employment | 0.88(0.81-0.95)^**^ | 0.88(0.74-1.06) | 1.16(1.11-1.20)^***^ | 0.89(0.84-0.95)^***^ | 0.97(0.93-1.03) | 0.89(0.80-0.98)^*^ | 1.05^†^(0.98-1.12) | 0.93(0.81-1.07) |
| Retire | 1.09(1.01-1.18)^*^ | 1.70(1.42-2.03)^***^ | 1.28(1.15-1.43)^***^ | 1.53(1.39-1.69)^***^ | 1.14(1.03-1.26)^*^ | 1.70(1.40-2.07)^***^ | 1.35^†^(1.15-1.58)^***^ | 1.53(1.30-1.81)^***^ |
| Others (student, etc.) | 0.73(0.67-0.80)^***^ | 1.29(1.07-1.56)^**^ | 1.03(0.97-1.09) | 0.86(0.82-0.91)^***^ | 0.89(0.86-0.92)^***^ | 1.37(1.22-1.52)^***^ | 1.08^†^(0.97-1.20) | 1.19(1.05-1.34)^**^ |
| **Household income per capita (ref=income level 1)** | | | | | | | | |
| Income level 2 | 1.37(1.27-1.47)^***^ | 1.24(1.15-1.34)^***^ | 1.06(0.94-1.20) | 1.05(0.95-1.16) | 1.17(1.08-1.26)^***^ | 1.01(0.93-1.11) | 1.15^†^(1.04-1.27)^**^ | 1.03(0.86-1.24) |
| Income level 3 | 1.50(1.36-1.65)^***^ | 1.28(1.18-1.39)^***^ | 1.02(0.93-1.11) | 0.98(0.90-1.08) | 1.03(0.95-1.11) | 0.87(0.80-0.96)^**^ | 1.20^†^(1.14-1.26)^***^ | 0.98^‡^(0.82-1.19) |
| Income level 4 | 1.76(1.57-1.98)^***^ | 1.86(1.66-2.08)^***^ | 1.07(0.95-1.19) | 1.39(1.22-1.59)^***^ | 1.20(1.13-1.29)^***^ | 0.80(0.61-1.03) | 1.30^†^(1.16-1.44)^***^ | 0.98^‡^(0.82-1.19) |
| **Insurance (ref=uninsured)** | | | | | | | | |
| GIS | 0.89(0.80-0.98)^*^ | 1.47(1.14-1.89)^**^ | 1.09(0.98-1.21) | 1.88(1.47-2.40)^***^ | 1.29(1.16-1.45)^***^ | 1.70(1.44-2.01)^***^ | 0.81(0.70-0.93)^**^ | 1.48(1.29-1.69)^***^ |
| UEBMI/LMI | 1.11(1.06-1.18)^***^ | 1.69(1.42-2.00)^***^ | 1.10(1.01-1.19)^*^ | 1.72(1.43-2.06)^***^ | 1.35(1.28-1.43)^***^ | 1.67(1.52-1.84)^***^ | 1.07(0.95-1.20) | 1.87(1.55-2.26)^***^ |
| URBMI | 0.93(0.90-0.96)^***^ | 1.18(0.98-1.41) | 1.24(1.10-1.40)^***^ | 1.33(1.03-1.72)^*^ | 1.58(1.47-1.70)^***^ | 1.37(1.24-1.52)^***^ | 1.12^†^(0.93-1.36) | 1.91^‡^(1.64-2.21)^***^ |
| NCMS | 1.31(1.20-1.43)^***^ | 0.97(0.82-1.13) | 1.28(1.09-1.51)^**^ | 1.26(1.02-1.55)^*^ | 1.48(1.38-1.58)^***^ | 1.17(1.05-1.31)^**^ | 1.04^†^(0.91-1.18) | 1.59^‡^(1.32-1.92)^***^ |
| CMI | 0.93(0.79-1.08) | 0.85(0.68-1.05) | 0.93(0.75-1.16) | 0.75(0.60-0.93)^**^ | 0.84(0.75-0.94)^**^ | 0.65(0.45-0.92)^*^ | 1.09(0.80-1.49) | 1.63^‡^(1.37-1.94)^***^ |
| **District (ref=western)** |  |  |  |  |  |  |  |  |
| Middle | 0.90(0.83-0.99)^*^ | 0.92(0.79-1.07) | 0.79(0.74-0.84)^***^ | 0.59(0.57-0.61)^***^ | 0.76(0.71-0.82)^***^ | 0.56(0.50-0.62)^***^ | 1.11^†^(1.03-1.20)^**^ | 0.85(0.71-1.01) |
| East | 0.95(0.85-1.07) | 1.39(1.03-1.90)^*^ | 0.88(0.74-1.06) | 1.30(1.09-1.56)^**^ | 0.88(0.81-0.96)^**^ | 1.26(1.03-1.54)^*^ | 1.15^†^(1.06-1.25)^**^ | 1.99(1.59-2.50)^***^ |
| **Travel time (ref=15+ Mins)** | | | | | | | | |
| <15 | - | - | 1.04(1.02-1.06)^***^ | 1.40(1.34-1.45)^***^ | 0.97(0.90-1.04) | 1.49(1.39-1.61)^***^ | 0.95^^(0.91-1.00)^*^ | 1.84^#^(1.72-1.96)^***^ |

^*^P<0.05; ^**^P<0.01; ^***^P<0.0001 (two-tailed test); **^§^**The odds ratios of age represent the change in the odds when the variable age is increased by ten years; ^†^The difference between the odd ratios of making 3–5 CHS visits in 2008 and 2011 was significant; ^‡^The difference between the odd ratios of making ≥6 visits in 2008 and 2011 was significant; ^^The difference between the odd ratios of making 3–5 CHS visits in 2009 and 2011 was significant; ^#^The difference between the odd ratios of making ≥6 visits in 2009 and 2011 was significant.

CHS=community health service, GMI=Government Medical Insurance, UEBMI= Urban Employee Basic Medical Insurance, URBMI=Urban Resident Basic Medical Insurance, LMI=Labor Medical Insurance, NCMS=New Cooperative Medical Scheme, CMI=Commercial Medical Insurance
