# Supplementary material for: Social Determinants of Community Health Services Utilization among the Users in China: A 4-Year Cross-Sectional Study
Source: PLoS One. 2014 May 22;9(5):e98095. doi: 10.1371/journal.pone.0098095 (PMC4031144; doi:10.1371/journal.pone.0098095)
Supplement: Table S2 — Multinomial logistic regressions for the association with frequency of CHS utilization among female CHS users. (DOC) [file pone.0098095.s002.doc]

**Table S2 Multinomial logistic regressions** for the association with frequency of CHS utilization among female CHS users

| **Variables** | **2008** | | **2009** | | **2010** | | **2011** | |
| --- | --- | --- | --- | --- | --- | --- | --- | --- |
|  | **3–5 visits** | **≥6 visits** | **3–5 visits** | **≥6 visits** | **3–5 visits** | **≥6 visits** | **3–5 visits** | **≥6 visits** |
| **Age^§^** | 1.09(1.06-1.13)^***^ | 1.26(1.22-1.30)^***^ | 1.11(1.08-1.14)^***^ | 1.33(1.31-1.34)^***^ | 1.11(1.07-1.14)^***^ | 1.24(1.19-1.30)^***^ | 1.10(1.07-1.12)^***^ | 1.23(1.20-1.26)^***^ |
| **Education (ref=primary school or below)** | | | | | | | | |
| Junior middle school | 0.91(0.83-1.00) | 0.86(0.81-0.91)^***^ | 0.95(0.89-1.02) | 0.90(0.83-0.98)^**^ | 0.96(0.92-1.01) | 1.09(1.00-1.18)^*^ | 1.06^†^(1.01-1.11)^*^ | 1.21^‡^(1.15-1.28)^***^ |
| Senior middle school | 0.85(0.79-0.93)^***^ | 0.79(0.77-0.81)^***^ | 0.96(0.90-1.03) | 0.85(0.78-0.93)^***^ | 1.01(0.98-1.05) | 1.21(1.11-1.31)^***^ | 1.07^†^(1.00-1.15)^*^ | 1.29^‡^(1.24-1.35)^***^ |
| College degree or above | 0.79(0.72-0.87)^***^ | 0.73(0.63-0.84)^***^ | 0.90(0.81-1.02) | 0.95(0.83-1.09) | 0.97(0.92-1.03) | 1.21(1.13-1.29)^***^ | 1.05^†^(1.00-1.10)^*^ | 1.44^‡^(1.34-1.56)^***^ |
| **Employment status (ref=unemployment)** | | | | | | | | |
| Employment | 1.12(1.01-1.25)^*^ | 1.02(0.91-1.15) | 0.90(0.82-0.98)^*^ | 0.89(0.85-0.94)^***^ | 0.99(0.90-1.09) | 0.59(0.53-0.65)^***^ | 1.25^†^(1.21-1.3)^***^ | 1.12(1.03-1.22)^**^ |
| Retire | 1.15(1.06-1.25)^***^ | 1.98(1.79-2.20)^***^ | 1.14(1.08-1.21)^***^ | 1.72(1.52-1.93)^***^ | 0.98(0.83-1.17) | 1.06(0.91-1.23) | 1.19(1.13-1.25)^***^ | 2.22(1.85-2.65)^***^ |
| Others (student, housewife) | 0.94(0.89-1.00) | 1.28(1.07-1.55)^**^ | 0.78(0.71-0.85)^***^ | 1.13(1.04-1.22)^**^ | 0.92(0.85-0.99)^*^ | 1.00(0.96-1.03) | 1.06(0.95-1.18) | 1.58(1.42-1.75)^***^ |
| **Household income per capita (ref=income level 1)** | | | | | | | | |
| Income level 2 | 1.26(1.18-1.34)^***^ | 1.04(0.95-1.15) | 1.28(1.19-1.38)^***^ | 1.20(1.11-1.30)^***^ | 0.90(0.80-1.02) | 0.95(0.77-1.17) | 1.06(0.89-1.27) | 0.98(0.86-1.12) |
| Income level 3 | 1.37(1.23-1.52)^***^ | 1.07(1.00-1.15)^*^ | 1.18(1.06-1.32)^**^ | 1.24(1.10-1.39)^***^ | 0.99(0.90-1.09) | 1.06(0.89-1.27) | 1.14(0.95-1.35) | 1.15(1.07-1.25)^***^ |
| Income level 4 | 1.35(1.21-1.51)^***^ | 1.36(1.22-1.51)^***^ | 1.30(1.20-1.41)^***^ | 1.58(1.48-1.68)^***^ | 1.00(0.86-1.16) | 1.01(0.76-1.34) | 1.06^†^(0.87-1.3) | 0.96^‡^(0.84-1.11) |
| **Insurance (ref=uninsured)** | | | | | | | | |
| GIS | 0.90(0.82-0.98)^*^ | 1.33(1.15-1.54)^***^ | 0.99(0.86-1.13) | 1.11(1.01-1.22)^*^ | 0.74(0.71-0.77)^***^ | 1.09(0.93-1.29) | 0.92(0.88-0.95)^***^ | 1.17(1.02-1.34)^*^ |
| UEBMI/LMI | 0.95(0.89-1.02) | 1.31(1.19-1.46)^***^ | 1.26(1.15-1.38)^***^ | 1.49(1.38-1.61)^***^ | 1.14(1.10-1.20)^***^ | 1.39(1.17-1.65)^***^ | 1.17^†^(1.13-1.22)^***^ | 1.61^‡^(1.47-1.76)^***^ |
| URBMI | 0.98(0.90-1.06) | 1.09(0.95-1.24) | 1.22(1.17-1.28)^***^ | 1.35(1.22-1.50)^***^ | 1.14(1.10-1.18)^***^ | 1.13(1.03-1.24)^**^ | 1.33^†^(1.28-1.38)^***^ | 1.58^‡^(1.48-1.69)^***^ |
| NCMS | 1.01(0.87-1.17) | 0.58(0.47-0.70)^***^ | 1.28(1.17-1.40)^***^ | 0.75(0.67-0.85)^***^ | 1.26(1.15-1.37)^***^ | 1.13(0.99-1.28) | 1.25^†^(1.17-1.34)^***^ | 1.33^‡^(1.25-1.42)^***^ |
| CMI | 0.92(0.85-1.00) | 1.21(1.00-1.47) | 0.88(0.74-1.04) | 0.47(0.37-0.60)^***^ | 0.65(0.53-0.79)^***^ | 1.20(0.91-1.57) | 1.29(0.92-1.81) | 1.56^‡^(1.33-1.83)^***^ |
| **District (ref=western)** | | | | | | | | |
| Middle | 1.25(1.11-1.41)^***^ | 0.95(0.73-1.24) | 0.92(0.88-0.97)^**^ | 0.69(0.67-0.70)^***^ | 0.80(0.77-0.84)^***^ | 0.58(0.54-0.63)^***^ | 1.10(1.01-1.20)^*^ | 0.73(0.61-0.88)^***^ |
| East | 0.99(0.87-1.14) | 1.67(1.14-2.43)^**^ | 1.11(0.97-1.28) | 1.54(1.20-1.98)^***^ | 1.04(0.96-1.12) | 1.65(1.35-2.01)^***^ | 1.13(1.04-1.23)^**^ | 1.87(1.48-2.37)^***^ |
| **Travel time (ref=15+ Mins)** | | | | | | | | |
| <15 | - | - | 1.00(0.96-1.05) | 1.23(1.08-1.39)^**^ | 0.99(0.94-1.05) | 1.25(1.21-1.30)^***^ | 0.91^^(0.88-0.95)^***^ | 1.64^#^(1.49-1.82)^***^ |

^*^P<0.05; ^**^P<0.01; ^***^P<0.0001 (two-tailed test); **^§^**The odds ratios of age represent the change in the odds when the variable age is increased by ten years; ^†^The difference between the odd ratios of making 3–5 CHS visits in 2008 and 2011 was significant; ^‡^The difference between the odd ratios of making ≥6 visits in 2008 and 2011 was significant; ^^The difference between the odd ratios of making 3–5 CHS visits in 2009 and 2011 was significant; ^#^The difference between the odd ratios of making ≥6 visits in 2009 and 2011 was significant.

CHS=community health service, GMI=Government Medical Insurance, UEBMI= Urban Employee Basic Medical Insurance, URBMI=Urban Resident Basic Medical Insurance, LMI=Labor Medical Insurance, NCMS=New Cooperative Medical Scheme, CMI=Commercial Medical Insurance
