# Supplementary material for: Social Determinants of Community Health Services Utilization among the Users in China: A 4-Year Cross-Sectional Study
Source: PLoS One. 2014 May 22;9(5):e98095. doi: 10.1371/journal.pone.0098095 (PMC4031144; doi:10.1371/journal.pone.0098095)
Supplement: Table S3 — Comparison of odds ratios of making 3–5 CHS visits in 2008 and 2011. (DOC) [file pone.0098095.s003.doc]

**Table S3 Comparison of odds ratios of making 3–5 CHS visits in** 2008 and 2011

| **Variables** | **3–5 CHS visits** | | |
| --- | --- | --- | --- |
|  | **2008** | **2011** | **Ratio of odd ratios** |
| **Gender (ref=male)** | 1.12(1.07-1.18)**^***^** | 1.11(1.10-1.13)**^***^** | 1.01(0.96-1.06) |
| **Age^§^** | 1.11(1.09-1.14)**^***^** | 1.11(1.09-1.12)**^***^** | 1.00(0.98-1.03) |
| **Education (ref=primary school or below)** |  |  |  |
| Junior middle school | 0.93(0.88-1.00)**^*^** | 1.09(1.05-1.14)**^***^** | 0.86(0.79-0.92) |
| Senior middle school | 0.88(0.79-0.97)**^**^** | 1.14(1.10-1.18)**^***^** | 0.77(0.69-0.86) |
| College degree or above | 0.81(0.75-0.88)**^***^** | 1.09(1.05-1.13)**^***^** | 0.74(0.68-0.81) |
| **Employment status (ref=unemployment)** |  |  |  |
| Employment | 1.00(0.96-1.05) | 1.16(1.12-1.21)**^***^** | 0.86(0.81-0.92) |
| Retire | 1.12(1.04-1.19)**^**^** | 1.26(1.17-1.36)**^***^** | 0.89(0.80-0.98) |
| Others (student, housewife) | 0.85(0.81-0.89)**^***^** | 1.07(0.98-1.16) | 0.80(0.72-0.88) |
| **Household income per capita (ref=income level 1)** |  |  |  |
| Income level 2 | 1.30(1.24-1.37)**^***^** | 1.10(1.00-1.20)**^*^** | 1.19(1.07-1.32) |
| Income level 3 | 1.42(1.29-1.55)**^***^** | 1.16(1.05-1.28)**^**^** | 1.22(1.07-1.40) |
| Income level 4 | 1.53(1.37-1.70)**^***^** | 1.16(1.03-1.31)**^*^** | 1.32(1.12-1.55) |
| **Insurance (ref=uninsured)** |  |  |  |
| GIS | 0.89(0.82-0.97)**^**^** | 0.87(0.82-0.93)**^***^** | 1.02(0.92-1.14) |
| UEBMI/LMI | 1.02(0.96-1.08) | 1.13(1.06-1.20)**^***^** | 0.90(0.83-0.98) |
| URBMI | 0.95(0.91-0.99)**^*^** | 1.24(1.16-1.33)**^***^** | 0.77(0.71-0.83) |
| NCMS | 1.13(1.03-1.24)**^**^** | 1.16(1.06-1.27)**^***^** | 0.97(0.86-1.11) |
| CMI | 0.93(0.86-1.00)**^*^** | 1.20(0.96-1.51) | 0.77(0.61-0.98) |
| **District(ref=western)** |  |  |  |
| Middle | 1.08(0.97-1.20) | 1.10(1.02-1.20)**^*^** | 0.98(0.86-1.12) |
| East | 0.97(0.86-1.10) | 1.13(1.04-1.23)**^**^** | 0.86(0.74-0.99) |
| **Travel time(ref=15+ Mins)**^†^ |  |  |  |
| <15 | 1.02(0.99-1.05) | 0.93(0.9-0.96)**^***^** | 1.10(1.05-1.15) |

**^§^**The odds ratios of age represent the change in the odds when the variable age is increased by ten years; ^†^Comparison of odds ratio of multinomial logistic regression in 2009 and 2011.

CHS=community health service, GMI=Government Medical Insurance, UEBMI= Urban Employee Basic Medical Insurance, URBMI=Urban Resident Basic Medical Insurance, LMI=Labor Medical Insurance, NCMS=New Cooperative Medical Scheme, CMI=Commercial Medical Insurance
