# Supplementary material for: Social Determinants of Community Health Services Utilization among the Users in China: A 4-Year Cross-Sectional Study
Source: PLoS One. 2014 May 22;9(5):e98095. doi: 10.1371/journal.pone.0098095 (PMC4031144; doi:10.1371/journal.pone.0098095)
Supplement: Table S4 — Comparison of odds ratios of making ≥6 CHS visits in 2008 and 2011. (DOC) [file pone.0098095.s004.doc]

**Table S4 Comparison of odds ratios of making ≥6 CHS visits** in 2008 and 2011

| **Variables** | **≥6 CHS visits** | | |
| --- | --- | --- | --- |
|  | **2008** | **2011** | **Ratio of odd ratios** |
| **Gender (ref=male)** | 1.37(1.28-1.47)^***^ | 1.27(1.25-1.30)^***^ | 1.08(1.00-1.15) |
| **Age^§^** | 1.33(1.30-1.35)^***^ | 1.29(1.26-1.32)^***^ | 1.03(1.00-1.06) |
| **Education (ref=primary school or below )** |  |  |  |
| Junior middle school | 0.94(0.90-0.99)^*^ | 1.26(1.21-1.30)^***^ | 0.75(0.70-0.80) |
| Senior middle school | 0.88(0.84-0.92)^***^ | 1.41(1.33-1.50)^***^ | 0.62(0.58-0.67) |
| College degree or above | 0.86(0.77-0.97)^*^ | 1.48(1.43-1.54)^***^ | 0.58(0.51-0.65) |
| **Employment status (ref=unemployment)** |  |  |  |
| Employment | 0.95(0.84-1.08) | 1.04(0.95-1.14) | 0.92(0.78-1.07) |
| Retire | 1.85(1.65-2.08)^***^ | 1.91(1.68-2.18)^***^ | 0.97(0.82-1.15) |
| Others (student, housewife) | 1.28(1.08-1.52)^**^ | 1.41(1.32-1.51)^***^ | 0.91(0.76-1.09) |
| **Household income per capita (ref=income level 1)** |  |  |  |
| Income level 2 | 1.11(1.03-1.20)^**^ | 1.00(0.97-1.03) | 1.11(1.02-1.21) |
| Income level 3 | 1.15(1.09-1.21)^***^ | 1.08(0.99-1.17) | 1.06(0.96-1.18) |
| Income level 4 | 1.55(1.42-1.69)^***^ | 0.97(0.86-1.09) | 1.60(1.38-1.84) |
| **Insurance (ref=uninsured)** |  |  |  |
| GIS | 1.38(1.17-1.63)^***^ | 1.27(1.13-1.43)^***^ | 1.08(0.89-1.32) |
| UEBMI/LMI | 1.45(1.29-1.62)^***^ | 1.68(1.49-1.90)^***^ | 0.86(0.73-1.01) |
| URBMI | 1.11(0.96-1.28) | 1.67(1.53-1.82)^***^ | 0.66(0.56-0.78) |
| NCMS | 0.72(0.61-0.84)^***^ | 1.42(1.30-1.54) ^***^ | 0.51(0.42-0.60) |
| CMI | 1.11(0.91-1.35) | 1.55(1.33-1.80)^***^ | 0.71(0.56-0.91) |
| **District(ref=western)** |  |  |  |
| Middle | 0.94(0.76-1.16) | 0.78(0.66-0.94)^**^ | 1.20(0.91-1.58) |
| East | 1.55(1.11-2.16)^*^ | 1.92(1.53-2.42)^***^ | 0.81(0.54-1.21) |
| **Travel time(ref=15+ Mins)**^†^ |  |  |  |
| <15 | 1.30(1.19-1.41)^***^ | 1.72(1.61-1.84)^***^ | 0.75(0.68-0.84) |

^§^The odds ratios of age represent the change in the odds when the variable age is increased by ten years; ^†^Comparison of odds ratio of multinomial logistic regression in 2009 and 2011.

CHS=community health service, GMI=Government Medical Insurance, UEBMI= Urban Employee Basic Medical Insurance, URBMI=Urban Resident Basic Medical Insurance, LMI=Labor Medical Insurance, NCMS=New Cooperative Medical Scheme, CMI=Commercial Medical Insurance
